# A Cross “Ethnical” Comparison of the Driver Behaviour Questionnaire (DBQ) in an Economically Fast Developing Country: DBQ in Arab Gulf and South Asian countries

**DOI:** 10.5539/gjhs.v5n4p165

**Published:** 2013-05-12

**Authors:** Abdulbari Bener, Mohamud Verjee, Elnour E. Dafeeah, Mohammad T. Yousafzai, Sundus Mari, Ahmed Hassib, Hamza Al-Khatib, Min Kyung Choi, Noor Nema, Türker Özkan, Timo Lajunen

**Affiliations:** 1 Dept. of Medical Statistics & Epidemiology, Hamad Medical Corporation & Dept. of Public Health, Weill Cornell Medical College, Qatar; 2 Dept. of Evidence for Population Health Unit, School of Epidemiology and Health Sciences, The University of Manchester, Manchester, UK; 3 Dept. of Medical Education Weill Cornell Medical College, Qatar; 4 Dept. of Psychiatry, Rumeilah Hospital, Hamad Medical Corporation, Qatar; 5 Safety Research Unit, Department of Psychology, Middle East Technical University, Ankara Turkey

**Keywords:** Driver Behaviour Questionnaire (DBQ), traffic accidents, ethnic groups group, Qatar, Jordan, Indian-sub-continental, Philippine

## Abstract

**Aim:**

The aim of this study was to compare the driving behaviours of four ethnic groups and to investigate the relationship between violations, errors and lapses of DBQ and accident involvement in Qatar.

**Subjects and Methods:**

The Driver Behaviour Questionnaire (DBQ) was used to measure the aberrant driving behaviours leading to accidents. Of 2400 drivers approached, 1824 drivers agreed to participate (76%) and completed the driver behaviour questionnaire and background information.

**Results:**

The study revealed that the majority of the Qatari (35.9%) and Jordanian drivers (37.5%) were below 30 years of age, whereas Filipino (42.3%) and Indian subcontinent (34.1%) drivers were in the age group of 30-39 years. Qatari drivers (52%) were involved in most accidents, followed by Jordanians (48.3%). The most common type of collision was a head on collision, which was similar in all four ethnic groups. The Qatari drivers scored higher on almost all items of violations, errors and lapses compared to other ethnic groups, while Filipino drivers were lower on all the items. The most common violation was the same in all four ethnic groups “Disregard the speed limits on a motorway”. The most common error item observed was “Queing to turn right/left on to a main road”. “Forget where you left your car” and “Hit something when reversing” were the two lapses identified in factor analysis.

**Conclusion:**

The present study identified that Qatari drivers scored higher on most of the items of violations, errors and lapses of DBQ compared to other countries, whereas Filipino drivers scored lower in DBQ items.

## 1. Introduction

Traffic collisions were reported as one of the 10 leading causes of death and are projected to become the third leading cause of disability adjusted life years (DALYs) lost by 2020 (Peden, McGee, & Krug, 2002). In the world, 50 million people are seriously injured and 1.5 million deaths are occurred in road traffic accidents (Peden et al., 2004). The most important variables in traffic safety appeared to be economic, societal and cultural factors. The state of Qatar, for example, had to go through a rapid transition in its socio-economic status during the last two decades. There was a sudden growth in population in rich developing countries including many different nationalities and ethnic and/or immigrant groups which also leads to an increase in vehicle demand. Studies conducted in Qatar (Bener, Crundall, Haigney, Bensiali, & Al-Falasi, 2007; Bener & Crundall, 2008; Bener, Ozkan, & Lajunen, 2008; Bener, Crundall, Özkan, & Lajunen, 2010) showed an increasing trend in road traffic accidents and different types of driver behaviour causing accidents among Qatari drivers. The findings of the previous studies (Bener et al., 2007; Bener & Crundall, 2008) have also shown that self-reported driving behaviours are associated with both active and passive traffic accidents among other drivers in different countries. It should also be noted that the significant relationship between self-reported driver behaviours (e.g., violations) and objective measures of highway driving has been recently reported (Zhao et al., 2012).

The Manchester Driver Behaviour Questionnaire (DBQ) (Reason, Manstead, Stradling, Baxter, & Campbell, 1990) is one of the most widely used instruments in Traffic Psychology for measuring self-reported driving style and investigating the relationship between driving behaviour and accident involvement (Dde Winter and Dodou, 2010, report 174 studies using some version of the DBQ). The DBQ questionnaire has three components; violations, errors and lapses (Parker, McDonald, Rabbitt, & Sutcliffe, 2000). Errors and violations can lead to death because they are potentially dangerous. In particular, violations have been reported to be associated with active loss-of-control and passive right-of-way accidents as well as with speeding and parking offences (Mesken, Lajunen, & Summala, 2002).

A considerable regional difference between countries was observed in road traffic accidents. Driver behaviour might vary from country to country because of the high number of potential interpersonal conflicts areas in these countries’ traffic environments. It was reported that, for example, there were significant differences between Western/Northern European and Southern European/Middle Eastern drivers especially in aggressive violations (Lajunen, Parker, & Strackling, 1998a; Lajunen, Corry, Summala, & Hartley, 1998b). It is well known that there are considerable differences between countries in driving style (Ozkan, Lajunen, Chliaoutakies, Parker, & Summala, 2006). However, the driving style of different ethnic groups in a country has remained unexamined in literature. The present study is, therefore, the first study aimed at investigating the cross “ethnical” applicability of the three-factor structure (violations, errors and lapses) of the Driver Behaviour Questionnaire (DBQ), comparing the driving behaviours of four ethnic groups (i.e., Qataris as a host group, Jordanians, Indians, and Philippines as immigrant groups) and investigating the relationship between the three factors of DBQ and accident involvement.

## 2. Methodology

### 2.1 Participants

Hamad Medical Corporation Institutional Review Board (IRB) and Weill Cornell Medical College (IRB) have given the approval for conducting this research. Qualified Nurses were trained to conduct face to face interview to complete the questionnaires. A randomly selected 2400 drivers during the period from January 2012 to September 2012 participated in this study. In the State of Qatar, there are 21 primary health centers located across the country with inhabitants equally distributed around every health center. Multistage stratified cluster sampling was applied to select the health centers and the study subjects were recruited from the patients and visitors attending these health centers.

A cross-sectional survey was conducted among Qatari, Jordanian, Indian sub-continental and Filipino drivers. We have sampled the study subjects from each region in a way that the sample size in each region was proportional to its share of total population in four countries. According to sample size, 2,400 drivers aged 18 years and above were recruited for face to face interview. 1824 drivers (471 Qataris, 445 Jordanians, 461 Indians, and 447 Filipinos out of total 2400 (600 each ethnicity) participated in this survey giving a response rate of 76%. Among the study sample, 48.4% of them were involved in at least one traffic accident. We have assured them that their information would be kept of anonymity and confidentiality. Furthermore, drivers were asked about socio-demographic characteristics.

### 2.2 Aberrant Driver Behaviours

Driver Behaviour Questionnaire (DBQ) with extended violations was used to measure aberrant driver behaviours. The DBQ questionnaire includes 10 items of ordinary violations, 8 items of lapses, and 8 items of errors. The DBQ questionnaire has 26 behaviours on a six-point scale (0 = never, 1= hardly ever, 2 = occasionally, 3 = quite often, 4 = frequently, and 5 = nearly all the time) and the research assistants asked the participants to indicate how often they have committed every behaviour in the previous year.

### 2.3 Statistical Analysis

Statistical Package for Social Sciences (SPSS version # 20) was used to analyze the data. Chi-square and Fisher's exact test (two-tailed) analysis were performed to test for differences in proportions of categorical variables between two or more groups. Analysis of variance (ANOVA) with pair wise post hoc Bonferroni correction was used to identify differences in tendency to commit aberrant driving behaviors across the four ethnic groups after controlling for age, gender and mileage driven since obtaining their driving licenses. Principal component analysis (PCA) with Varimax rotation and Kaiser Normalization were performed to examine the factor structure of the DBQ among Qataris, Jordanians, Indian Sub-continent and Filipino. Internal consistencies of each factor with percentage variance were calculated using Cronbach's alpha coefficients. The level p<0.05 was considered as the cut-off value for significance.

## 3. Results

### 3.1 Participants’ Profile

Table 1 compares the socio-demographic variables of the studied drivers among four different ethnic groups. Majority of the Qatari (35.9%) and Jordanian drivers (37.5%) were below 30 years of age, whereas Filipino (42.3%) and Indian sub continental (34.1%) drivers were in the age group of 30 – 39 years with a significant difference (p<0.001). Overall, 74.6% of the studied drivers were men and 25.4% women. Filipino drivers had significantly higher education with university degree (59.5%) as compared to other ethnic groups with secondary education; Qatari (35.2%), Jordanian (35.3%) and Indian sub continental (33.8%) (p<0.001). More than half of the drivers of the four ethnic groups were holding professional jobs with a higher proportion among Filipino drivers (79%). More than 10 years of experience was significantly higher among drivers of Indian sub continents (40.6%) and Philippines (42.1%) (p=0.02). A significant difference was observed across the four ethnic groups in terms of age group (p<0.001), education (p<0.001), occupation (p<0.001), marital status (p<0.001), driving experience (p=0.02) and annual mileage (p<0.001).

**Table 1:** Comparison of socio-demographic characteristics of the studied drivers among four different ethnic groups (N=1824)

Variables	Total N=1824	Qatari n=471 n (%)	Jordanian n=445 n (%)	Indian-Sub Continent n=461 n (%)	Filipino n=447 n (%)	P value
Age Group						<0.001
<30	575(31.5)	169(35.9)	167(37.5)	149(32.3)	90(20.1)	
30-39	639(35.0)	162(34.4)	131(29.4)	157(34.1)	189(42.3)	
40-49	415(22.8)	93(19.7)	95(21.3)	113(24.5)	114(25.5)	
≥50	195(10.7)	47(10.0)	52(11.7)	42(9.1)	54(12.1)	

Gender						0.738
Male	1359(74.5)	360(76.4)	329(73.9)	344(74.6)	326(72.9)	
Female	465(25.5)	111(23.6)	116(26.1)	117(25.4)	121(27.1)	

Education						<0.001
Illiterate	166(9.1)	54(11.5)	49(11.0)	55(11.9)	8(1.8)	
Primary	312(17.1)	100(21.2)	88(19.8)	95(20.6)	29(6.5)	
Intermediate	284(15.6)	69(14.6)	63(14.2)	69(15.0)	83(18.6)	
Secondary	540(29.6)	166(35.2)	157(35.3)	156(33.8)	61(13.6)	
University	522(28.6)	82(17.4)	88(19.8)	86(18.7)	266(59.5)

Occupation						<0.001
Student/ Not working	188(10.3)	67(14.2)	66(14.8)	36(7.8)	19(4.3)	
Housewife	145(7.9)	39(8.3)	39(8.8)	48(10.4)	19(4.3)	
Professional	1170(64.1)	276(58.6)	253(56.9)	288(62.5)	353(79.0)	
Manual/drivers	321(17.6)	89(18.9)	87(19.6)	89(19.3)	56(12.5)	

Marital status						<0.001
Single	322(17.7)	115(24.4)	87(19.6)	63(13.7)	57(12.8)	
Married	1434(78.6)	340(72.2)	336(75.5)	374(81.1)	384(85.9)	
Divorce/Widowed	68(3.7)	16(3.4)	22(4.9)	24(5.2)	6(1.3)	

Driving experience						0.022
<2 year	242(13.3)	60(12.7)	52(11.7)	58(12.6)	72(16.1)	
2-5 years	477(26.2)	122(25.9)	133(29.9)	129(28.0)	93(20.8)	
5-10 years	400(21.9)	118(25.1)	101(22.7)	87(18.9)	94(21.0)	
>10 years	705(38.7)	171(36.3)	159(35.7)	187(40.6)	188(42.1)	

Monthly income*:						0.426
<10,000	632(34.6)	171(36.3)	163(36.6)	149(32.3)	149(33.3)	
≥10,000	1192(65.4)	300(63.7)	282(63.4)	312(67.7)	298(66.7)	

Place of living:						0.299
Urban	1290(70.7)	334(70.9)	303(68.1)	323(70.1)	330(73.8)	
Semi-urban	534(29.3)	137(29.1)	142(31.9)	138(29.9)	117(26.2)	

Car Type						0.094
4WD	762(41.8)	199(42.3)	171(38.4)	185(40.1)	207(46.3)	
Small Car	1062(58.2)	272(57.7)	274(61.6)	276(59.9)	240(53.7)	

Annual mileage	33954±21189	37381±20920	32370±22218	34531±22479	31323±18418	<0.001

Seat Belt use						0.859
Yes	1133(62.1)	292(62.0)	277(62.2)	280(60.7)	284(63.5)	
No	691(37.9)	179(38.0)	168(37.8)	181(39.3)	163(36.5)	

* Qatari Riyal. (1US$ =3.64Qatari Riyal)

### 3.2 Road Traffic Accidents and Nature of Collision

Table 2 compares the road traffic accident characteristics and nature of collision among studied drivers of four ethnic groups. Qatari drivers (52%) were involved in more accidents, followed by Jordanians (48.3%) and Indian Sub continentals (48.2%). In terms of cause of accidents, careless driving (49%) and excessive speeding (44.4%) were significantly higher among Qatari drivers (p<0.001). More than one third of the studied drivers of four ethnic groups were involved in traffic violations; Qatari (46.5%), Jordanian (37.8%), Indian Sub Continentals (37.3%) and Filipinos (32.9%). Most of the accidents took place near roundabouts for Qataris (49.8%), Jordanians (41.4%) and Indian sub continentals (49.5%), while a significant number of accidents took place on the main road for Filipinos drivers (54.7%) (p<0.001). The most common type of collision was a head on collision which was similar in all four countries. The second most common collision, overturn skid, was similar in Jordan (14.9%) and Qatar (18%), whereas angle collision was more frequent in Indian subcontinent (14.4%) and Philippines (18.4%).

**Table 2:** Comparison of road traffic characteristics and nature of collision among Surveyed drivers in Qatar (N=1824)

Variables	Total N=1824(%)	Qatari n=471(%)	Jordanian n=445(%)	Indian-Sub Continent n=461(%)	Filipino n=447(%)	P value
Ever had Accident						0.204
Yes	883(48.4)	245(52.0)	215(48.3)	222(48.2)	201(45.0)
No	941(51.6)	226(48.0)	230(51.7)	239(51.8)	246(55.0)
Cause of Accident: §						
Careless driving	641(35.1)	231(49.0)	134(30.1)	141(30.6)	138(30.9)	<0.001
Excessive Speeding	658(36.1)	209(44.4)	173(38.9)	186(40.3)	90(20.1)	<0.001
Alcohol/Drug	156(8.6)	15(3.2)	9(2.0)	10(2.2)	122(27.3)	<0.001
Traffic violation	535(29.3)	219(46.5)	168(37.8)	172(37.3)	23(32.9)	0.876

Injury (only among those who had accident N=883)	n=883	n=245	n=215	n=222	n=201	0.001
Yes	702(79.5)	216(88.2)	153(71.2)	174(78.4)		
No	181(20.5)	29(11.8)	62(28.8)	48(21.6)		
Accident Location						<0.001
Main Road	322(36.5)	66(26.9)	72(33.5)	74(33.3)	
Side Road	166(18.8)	29(11.8)	40(18.6)	19(8.6)	
At the crossroad	63(7.1)	28(11.4)	14(6.5)	19(8.6)	
Roundabout	332(37.6)	122(49.8)	89(41.4)	110(49.5)	11(5.5)
Nature of collision						<0.001*
Pedestrian	122(13.8)	22(9.0)	28(13.0)	26(11.7)	46(22.9)
Head on collision	225(25.5)	49(20.0)	55(25.6)	56(25.2)	65(32.3)
Angle collision	126(14.3)	31(12.7)	26(12.1)	32(14.4)	37(18.4)
Rear end hit	82(9.3)	29(11.8)	21(9.8)	24(10.8)	8(4.0)
Nose to tail	68(7.7)	23(9.4)	13(6.0)	28(12.6)	4(2.0)
Side collision	47(5.3)	13(5.3)	10(4.7)	9(4.1)	15(7.5)
Hit parked vehicle	21(2.4)	7(2.9)	6(2.8)	8(3.6)	0
Hit fixed object	45(5.1)	20(8.2)	11(5.1)	7(3.2)	7(3.5)
Overturn skid	107(12.1)	44(18.0)	32(14.9)	27(12.2)	4(2.0)
Crash road sign	40(4.5)	7(2.9)	13(6.0)	5(2.3)	15(7.5)

§ Multiple responses, number and percentages may not sum up to total sample.

* The p value based Chi Square test may not be valid due to “0” count in one cell.

### 3.3 Driver Behaviour Questionnaire (DBQ)

Table 3 shows the mean scores for each of the individual items relating to violations, errors and lapses in the driver behaviour questionnaire among drivers in four different ethnic groups. Qatari drivers scored higher on all violation items with a significant difference in comparison to the other three ethnic groups. The two most common violations were similar in Qatari (2.29 & 2.21), Jordanian (1.82 & 1.78) and Filipinos (1.45 & 1.45) drivers which were more impatient with a slow driver. Hit the car in front was the most frequent error among Qataris (1.66) and Indian sub continental drivers (1.57). Also turning right/left nearly hit a two wheeler” was the most common error among Jordanians and overtaking was most common among Filipino drivers. Across the four ethnic groups, Filipino drivers had the lowest mean score for all error items and they were significantly different from other ethnic groups.

**Table 3:** Means and standard deviations of items of Driver Behaviour Questionnaire (DBQ) among drivers in four different ethnic groups (N=1824)

Variables	Qatari n=471	Jordanian n=445	Indian-Sub Continent n=461	Filipino n=447
**Violations**				
Drive especially close to the car in front as a signal to its driver to go faster or get out of the way	1.75 (1.76)*	1.29(1.49)	1.54(1.50)	1.11(1.32)§
Cross a junction knowing that the traffic lights have already turned red	1.49(1.63)*	1.12(1.47)	1.31(1.49)	0.54(1.10)*
Disregard the speed limits late at night or early in the morning	2.10(1.77)*	1.53(1.59)*	1.88(1.65)*	1.00(1.29)*
Disregard the speed limits on a motorway	1.74(1.72)*	1.38(1.61)	1.52(1.53)	1.02(1.34)*
Have an aversion to a particular class of road user and indicate your hostility by whatever means you can	1.53(1.62)*	1.06(1.42)	1.33(1.40)	1.07(1.17)§
Become impatient with a slow driver in the outer lane and overtake on the inside (right) lane	2.29(1.84)*	1.82(1.74)	1.87(1.70)	1.45(1.36)*
Get involved with unofficial ‘races’ with other drivers	1.89(1.76)*	1.29(1.54)*	1.59(1.53)*	0.73(1.15)*
Angered by another driver’s behaviour, you give chase with the intention of giving him/her a piece of your mind	1.84(1.65)*	1.33(1.48)	1.30(1.36)	1.12(1.35) ‡
Sound your horn to indicate your annoyance to another driver	2.21(1.67)	1.78(1.57)*	2.01(1.64)	1.45(1.27)*
Stay in a motorway that you know will be closed ahead until the last minute before forcing you way into the other lane	1.55(1.52)*	1.26(1.57)	1.27(1.41)	1.10(1.28)
**Errors**				
Attempt to overtake someone that you hadn’t noticed to be signaling a left/right turn	1.23(1.49)	1.22(1.53)	1.38(1.68)	1.01(1.15)*
Miss ‘Give Way’ signs and narrowly avoid colliding with traffic having right of way	1.42(1.51)	1.13(1.49)*	1.39(1.59)	0.84(1.08)*
Fail to notice that pedestrians are crossing when turning into a side street from a main road	1.35(1.44)	1.18(1.43)	1.39(1.50)	0.78(1.10)*
Queuing to turn right/left onto a main road, you pay such close attention to the mainstream of traffic that you nearly hit the car in front	1.66(1.59)	1.35(1.58)*	1.57(1.69)	1.01(1.88)*
On turning right/left nearly hit a two wheeler who has come up on your inside	1.57(1.54)	1.38(1.50)	1.47(1.50)	0.84(1.28)*
Fail to check your rear-view mirror before pulling out or changing lanes, etc	1.43(1.38)	1.26(1.34)	1.39(1.42)	1.01(1.29)*
Under estimate the speed of an oncoming vehicle when overtaking	1.53(1.43)	1.22(1.40)§	1.52(1.48)	1.10(1.31)§
Apply sudden brakes on a slippery road, or steer wrong way in a skid	1.41(1.27)	1.28(1.30)	1.32(1.25)	0.94(1.24)*
**Lapses**				
Get into the wrong lane when approaching a roundabout or a junction	1.09(1.36)	0.85(1.17)§	1.11(1.47)	0.84(1.12)§
Misread the signs and exit from the roundabout on the wrong road	1.47(1.71)	1.33(1.71)	1.43(1.69)	0.76(1.09)*
Forget where you left your car in the car park	1.14(1.30)	1.02(1.27)	1.06(1.27)	1.17(1.23)
Hit something when reversing that you had not previously seen	1.57(1.38)*	1.37(1.29)	1.39(1.36)	1.15(1.21)*
Attempt to drive away from the traffic lights	1.65(1.41)	1.36(1.44)*	1.54(1.44)	1.04(1.24)*
Switch on one thing, such as headlights, when you meant to switch on something else, such as wipers	1.74(2.17)	1.50(1.66)^¥^	1.77(1.75)	1.17(2.38)*
Intending to drive to destination A and, you ‘wake up’ to find yourself in destination B, because the latter is your more usual destination	1.39(1.42)	1.19(1.39)§	1.34(1.45)	1.10(1.13)§
Realize you have no clear recollection of the road along which you have been travelling	1.44(1.56)	1.22(1.44)*	1.46(1.61)	0.91(1.14)*

All the numbers are presented as Mean (SD);

* significantly different from other three ethnic groups (p<0.05).

§ significant difference from Qatari and Indians (p<0.05).

‡ significant difference from Qatari and Jordanians (p<0.05).

¥ significantly different from Filipino and Indians (p<0.05).

### 3.4 Principal Component Analysis of DBQ and Reliability Coefficients

Table 4 shows the three factor solution of DBQ items including violations, errors and lapses over the four countries. The factor included ten items of violations in the Qatari sample, nine in the Filipino sample, seven in the Jordanian sample and eight in the Indian sample. “Disregard the speed limits on a motorway”, “disregard the speed limit late at night and early in the morning”, “cross a junction knowing that the traffic lights have already turned red” and “drive especially close to the car in front as a signal to its driver to go faster” were the items which loaded highest on violation in four countries. The factor included five items of errors in the Qatari sample, while the other countries had only 4 items. “Miss give way signs and narrowly avoid colliding with traffic having right of way” and “fail to notice that pedestrians are crossing when turning into a side street from a main road” were the two error items which loaded highest on errors in Qatar and Jordan. The third component included six items of lapses in Indian sample, five items in the Qatar and Jordan samples and four items in Filipino sample.

**Table 4:** Three-factor solution of the DBQ items, Cronbach’s alpha coefficients, and variance of the DBQ subscales across different ethnic groups in Qatar (N=1824)

		Errors				Violations				Lapses			
		
		Qa	Jo	Ind	Phil	Qa	Jo	Ind	Phil	Qa	Jo	Ind	Phil
Ov	Drive especially close to the car in front as a signal to its driver to go faster or get out of the way					.62	.62	.50	.62				
Ov	Cross a junction knowing that the traffic lights have already turned red					.60	.61	.47	.67				
Ov	Disregard the speed limits late at night or early in the morning					.64	.46	.45	.79				
Ov	Disregard the speed limits on a motorway					.72	.76	.67	.78				
Ov	Stay in a motorway that you know will be closed ahead until the last minute before forcing you way into the other lane					.55	-	-	-				
Ov	Become impatient with a slow driver in the outer lane and overtake on the inside (right) lane					.62	.74	.67	.69				
Ov	Get involved with unofficial ‘races’ with other drivers					.69	-	.61	.65				
Av	Angered by another driver’s behavior, you give chase with the intention of giving him/her a piece of your mind					.66	-	.58	.62				
Av	Sound your horn to indicate your annoyance to another driver					.60	.50	-	.52				
Av	Have an aversion to a particular class of road user and indicate your hostility by whatever means you can					.57	.49	.41	.56				
E	Miss ‘Give Way’ signs and narrowly avoid colliding with traffic having right of way	.46	.46	-	-								
E	Fail to notice that pedestrians are crossing when turning into a side street from a main road	.68	.49	-	-								
E	Queuing to turn right/left onto a main road, you pay such close attention to the mainstream of traffic that you nearly hit the car in front	.55	.61	.65	.64								
E	On turning right/left nearly hit a two wheeler who has come up on your inside	.57	-	-	.68								
E	Fail to check your rear-view mirror before pulling out or changing lanes, etc	-	.46	.47	.65								
E	Under estimate the speed of an oncoming vehicle when overtaking	.46	-	.48	-								
E	Apply sudden brakes on a slippery road, or steer wrong way in a skid	-	-	.52	.71								
E	Attempt to overtake someone that you hadn’t noticed to be signaling a left turn	-	-	-	-	-	-	-	-	-	-	-	-
L	Get into the wrong lane when approaching a roundabout or a junction									-	-	.42	.41
L	Misread the signs and exit from the roundabout on the wrong road									-	-	-	.56
L	Forget where you left your car in the car park									.75	.60	.77	.74
L	Hit something when reversing that you had not previously seen									.42	.67	.76	.74
L	Attempt to drive away from the traffic lights									.44	.56	.43	-
L	Switch on one thing, such as headlights, when you meant to switch on something else, such as wipers									-	.57	.51	-
L	Intending to drive to destination A and, you ‘wake up’ to find yourself in destination B, because the latter is your more usual destination									.46	-	-	-
L	Realize you have no clear recollection of the road along which you have been travelling									.42	.41	.32	-
	Eigen values	5.3	3.1	1.3	1.3	5.2	2.2	1.5	1.4	3.6	1.9	1.6	1.4
	Cronbach’s alpha	.65	.65	.58	.73	.81	.62	.51	.85	.65	.63	.57	.63
	Variance (%)	24	22	15	18	81	25	19	49	20.6	20.5	29.9	10.5

Principal component analysis; Varimax rotation with Kaiser Normalization. Factors extracted based on Eigen value>1. (Factor loadings below. 30 were omitted for the sake of clarity). Qa=Qatari, Jo=Jordanians, Ind.=Indian sub-continent, Phil.=Filipinos Av=aggressive violation, ov=ordinatry violations, e=error, L=lapse

## 4. Discussion

Road traffic related crashes impose an enormous public health burden globally. The present study has evaluated the key items of violations, errors and lapses which rated differently by Qatari, Jordanian, Indian, and Philippine drivers in Qatar. The data showed that Qatari drivers reported higher number of violations, errors and lapses, followed by Indian sub continental drivers, as compared to Jordanian and Filipino drivers. There was a significant difference observed between Qatari drivers and those of other ethnic groups. Even though the Qatari and Jordanian cultures show similarities, there is a significant difference observed in driver behaviour in both groups in Qatar. Qatari drivers, in general, appeared as the “riskiest group” and this is an interesting finding because immigrant groups in Europe, for example, are generally reported as “riskier” than “host” groups. Further studies should first investigate the topic among immigrants and host groups in other countries and then examine the social, cultural, and economical reasons of possible differences in the relationship between driver behaviour and accident involvement among different groups in a country and as compared to other countries.

Specifically, Qataris scored a higher mean score for violations especially for “become impatient with a slow driver in the outer lane and overtake on the right side (2.29) which is similar in Jordanian (1.82), Indian sub continental (1.87) and Filipino (1.45) drivers. “Sound your horn to indicate your annoyance to another driver” was the 2^nd^ most frequent violation item observed in four countries with a higher mean score among Qatari drivers (2.21), followed by Indian sub continental drivers (2.01). Jordanian (1.57) and Filipino drivers (1.45) reported this aggressive violation less frequently than Qatari and Indian drivers. The speed limit violations was the third key item of ordinary violations in Qatar (2.1), Jordan (1.53) and India (1.88), but in the Philippines (1.12) angered by another driver's behaviour and follow him” took the third place.

For errors, no significant difference was found between Qatari drivers and other drivers, whereas there was a significant difference observed in violations. The higher mean scores for two items of errors were similar among Qatari (1.66 & 1.57) and Jordanian drivers (1.35 & 1.38) with a higher mean score in Qatari drivers for turning right/left onto a main road. The Filipino drivers had the lowest mean score in almost all DBQ items. The possible reason for the lowest score is that nearly half of the Filipino drivers (42.1%) had more than 10 years driving experience. It was proposed in a study (Gregersen, 1996) that increasing driving experience and exposure to traffic increases the level of driving skills.

Similar to violations and errors, Qatari drivers reported lapses more frequently than drivers from the other three countries. The findings on lapses among Qatari drivers are similar to the results of Özkan et al. (2006) even though their study included different drivers from different countries.

The study also evaluated the differences in their socio-demographic and accident characteristics among these groups in Qatar. In the study sample, young drivers of the age group below 30 years among Qataris and Jordanians were involved with higher risk of having accidents whereas Filipino and Indian sub continent drivers were in the older age group 30 – 39 years with a significant difference. Young drivers are at a higher risk of crashing than older drivers because of their risk taking behaviour. Consistent with previous studies (Mesken et al., 2002; Bener et al., 2008), age was as an important factor in accident involvement. Also, there was a significant difference between the four ethnic groups in their age group. Among four groups, gender difference was similar in that male drivers reported road traffic accidents nearly four times as often as females which are similar to another study by Irershen (2004). These study findings are similar to the general finding in literature that greater accident risk is related to being male and a young driver (Elander, West, & French, 1993). This indicates that male and younger drivers were more prone to be involved in accidents among different ethnic groups in the same country as well.

The ordinary violation “Stay in a motor way… was loaded only in Qatar. It is worth noting that all of the drivers taking part in this study were involved with the violation “disregard the speed limit on a motor way. Similar to our study results, it was reported in a study that “stay in a motorway” was loaded highest on violations in Finland, Sweden and Turkey (Warner, Ozkan, Lajunen, & Tzamalouka, 2011).

Two lapses items identified in the factor analysis were “forget where you left your car in the car park” and “Hit something when reversing that you had not previously seen” which loaded highest in four countries. In Finland, Sweden and Turkey, different errors and lapses were loaded highest indicating the cultural difference (Warner et al., 2011). Özkan et al. (2006) reported that Finnish, British, Dutch, Greek and Turkish drivers had similarities and dissimilarities in driver behaviour and skills which is in accordance with the present research. Age, gender socio-economic and cultural differences between these samples could have caused the dissimilarities in factor structures found in this study.

Shinar (1998) reported that, for instance, the middle income level countries were more prone to interpersonal conflicts because of less developed infrastructure, lack of respect for rules and problems with enforcement. On the contrary, Qatar being a rich country took higher score on violations, errors and lapses compared to the high and middle income countries (Bener et al., 2007; Bener et al., 2008; De Winter, & Dodou, 2010). This shows that Qatar needs to improve the road infrastructure and reduce the traffic congestion which may cause aggressive driving. These results may indicate that different countries have different problems with regard to drivers’ aberrant behaviours and findings from one group are not necessarily applicable in other groups even in the same country. The different problems with regards to driver behaviours in each group need to be taken into account when promoting traffic safety interventions. The study suggests that driver behaviour has a significant effect on accidents and road safety awareness programs focusing on improving the behaviour of drivers can reduce the number of accidents.

## 5. Conclusion

The present study identified the key items of violations, errors and lapses which drivers from Qatar, Jordan, Indian sub continent and Philippine rated differently. The results indicate that behaviour choices are similar to each other with high score among Qatari drivers, but different from Jordanian and Filipino drivers, while Qatari and Indian Sub continent drivers’ responses resemble each other in most of the items. Qatari drivers reported more aggressive behaviours than the other drivers of the three countries. The factor analysis indicates that disregarding the speed limits, becoming impatient with slow drivers and sounding your horn to indicate your annoyance could be improved in every country.

### 5.1 Methodological Limitations

The data were based solely on drivers’ self-reports. However, several studies have indicated that self-reports of driving correspond well to actual driving behaviour. Some respondents may have underestimated the number of accidents in which they had been involved. Recently, it was found that there is a significant relationship between self-reported driver behaviours (e.g., violations) and objective measures of highway driving (Zhao et al., 2012).
